# Cytosolic 5′-Nucleotidase II Silencing in Lung Tumor Cells Regulates Metabolism through Activation of the p53/AMPK Signaling Pathway

**DOI:** 10.3390/ijms22137004

**Published:** 2021-06-29

**Authors:** Rossana Pesi, Simone Allegrini, Mercedes Garcia-Gil, Lucia Piazza, Roberta Moschini, Lars Petter Jordheim, Marcella Camici, Maria Grazia Tozzi

**Affiliations:** 1 Unità di Biochimica, Dipartimento di Biologia, Università di Pisa, Via San Zeno 51, 56127 Pisa, Italy; rossana.pesi@unipi.it (R.P.); l.piazza@studenti.unipi.it (L.P.); roberta.moschini@unipi.it (R.M.); marcella.camici@unipi.it (M.C.); maria.grazia.tozzi@unipi.it (M.G.T.); 2 Interdepartmental Research Center Nutrafood “Nutraceuticals and Food for Health”, Università di Pisa, 56126 Pisa, Italy; mercedes.garcia@unipi.it; 3 CISUP, Centro per l’Integrazione della Strumentazione dell’Università di Pisa, 56127 Pisa, Italy; 4 Unità di Fisiologia Generale, Dipartimento di Biologia, Università di Pisa, Via San Zeno 31, 56127 Pisa, Italy; 5 Université de Lyon, Université Claude Bernard Lyon 1, INSERM 1052, CNRS 5286, Centre Léon Bérard, Centre de Recherche en Cancérologie de Lyon, 69008 Lyon, France; lars-petter.jordheim@univ-lyon1.fr

**Keywords:** cN-II, p53, AMPK, lactate, metabolic regulation

## Abstract

Cytosolic 5′-nucleotidase II (cN-II) is an allosteric catabolic enzyme that hydrolyzes IMP, GMP, and AMP. The enzyme can assume at least two different structures, being the more active conformation stabilized by ATP and the less active by inorganic phosphate. Therefore, the variation in ATP concentration can control both structure and activity of cN-II. In this paper, using a capillary electrophoresis technique, we demonstrated that a partial silencing of cN-II in a pulmonary carcinoma cell line (NCI-H292) is accompanied by a decrease in adenylate pool, without affecting the energy charge. We also found that cN-II silencing decreased proliferation and increased oxidative metabolism, as indicated by the decreased production of lactate. These effects, as demonstrated by Western blotting, appear to be mediated by both p53 and AMP-activated protein kinase, as most of them are prevented by pifithrin-α, a known p53 inhibitor. These results are in line with our previous observations of a shift towards a more oxidative and less proliferative phenotype of tumoral cells with a low expression of cN-II, thus supporting the search for specific inhibitors of this enzyme as a therapeutic tool for the treatment of tumors.

## 1. Introduction

Stimulation of the expression and function of tumor suppressor proteins, as well as the inhibition of the expression or function of oncogenic proteins, represent a good approach to identify and develop new anticancer drugs [1,2,3,4]. The proteins involved in the tyrosine kinase receptor signaling pathway and mTOR are typically upregulated in cancer cells to sustain rapid cell growth, while onco-suppressors such as p53 and AMP-activated protein kinase (AMPK) are downregulated or mutated to prevent their function [1]. In healthy cells, the high proliferation rate requires signals of high energy charge, whereas a low energy charge activates AMPK and p53 to lower the rate of synthetic pathways and improve metabolic performance [5,6]. Therefore, these regulatory systems require precise information about the amount of ATP available for cellular processes at each moment. cN-II is an allosteric protein with at least two different structures—a highly active conformation, stabilized by interaction with ATP or other phosphorylated compounds such as diadenosine tetraphosphate (Ap4A), and a low activity conformation, which requires low ATP and high inorganic phosphate (P_i_) concentration to be stabilized. Structural analysis confirmed the existence of the two distinct conformations of cN-II obtained in the presence of allosteric effectors [7]. Oxidative stress causes the irreversible inactivation of the enzyme [8,9]. Therefore, K_M_ and k_cat_ of cN-II depend on the cell energy charge and on reactive oxygen species [8]. Many papers demonstrated that at a high energy charge, the cN-II activity contributes to the homeostasis of IMP, GMP and AMP either directly, since AMP is a poor substrate of the enzyme, or indirectly, hydrolyzing IMP derived from AMP deamination [8,10]. The combined action of AMP deaminase and cN-II, both activated by ATP, consents to eliminate the excess of purine ring as hypoxanthine or uric acid avoiding adenosine accumulation to occur. Adenosine is an important signal which influences a plethora of regulatory mechanisms through interaction with its receptors A1, A2a, A2b and A3 [11]. At a low energy charge, cN-II assumes a low activity conformation leading to IMP and AMP accumulation avoiding a dangerous depletion of purine nucleotide precursors [12]. In this condition, adenosine can be generated by AMP hydrolysis mediated by cN-I, an isoenzyme specific for AMP which is activated by ADP [10,13]. We have previously demonstrated that cN-II interacts with Ice protease-activating factor (IPAF), a protein involved in the innate immunity and inflammation, only in the conformation determined by high energy charge [9,14]. In addition, using different techniques, more than 40 different proteins are expected to interact with cN-II, such as proteins involved in the regulation of protein degradation and synthesis, transcription factors, tyrosine kinase receptors and their regulators (for a better insight see the following databases BioGRID; IntAct-EMBL-EBI). The high number of proteins able to interact with cN-II, the extremely high conservation of its structure during evolution, and the ubiquitous low expression level, made attractive the hypothesis that this enzyme might possess roles independent of its catalytic activity. Curiously, a partial silencing or a moderate over-expression of cN-II which simulate the physiological fluctuation between high and low enzyme activity, caused very evident effects on proliferation and metabolism [10,15,16]. Our results on tumoral cell models in which cN-II was partially silenced demonstrated that low activity correlates with a more oxidative phenotype, with low rate of protein synthesis, high mitochondrial content, high antioxidant defense, with a concomitant activation of p53, but the causal relationship was not demonstrated [8]. Furthermore, in a mice model, the knockout of the enzyme was associated with reduced body weight also after a high-fat diet [17]. Consistently, a single-nucleotide polymorphism in *NT5C2* (the cN-II gene) was associated with a lower tendency to accumulate adipose tissue in Japanese women [18,19]. Finally, recent reports describe several effects of cN-II silencing, possibly through activation of AMPK both in skeletal muscle and in human neural progenitor cells [20,21]. In this paper, we report the effects of partial cN-II silencing in a model of lung cancer (NCI-H292), demonstrating that cN-II silencing increases oxidative metabolism and decreases proliferation by activating both p53 and AMPK. The cell models were silenced for cN-II by stable transfection of shRNA-encoding plasmids as earlier described [22].

## 2. Results

### 2.1. cN-II Silencing Affects Nucleotide Content in NCI-H292 Cells

The specific activity of cN-II was measured in crude extracts of NCI-H292 cells transfected with a control plasmid (pScont cells) or a cN-II-targeting shRNA plasmid (pScNII cells). As expected, cN-II activity in pScNII cells was approximately one third of that of control cells (pScont). The presence of pifithrin-α in the growth medium did not affect significantly the enzyme activity (Table 1).

**Figure 1:**
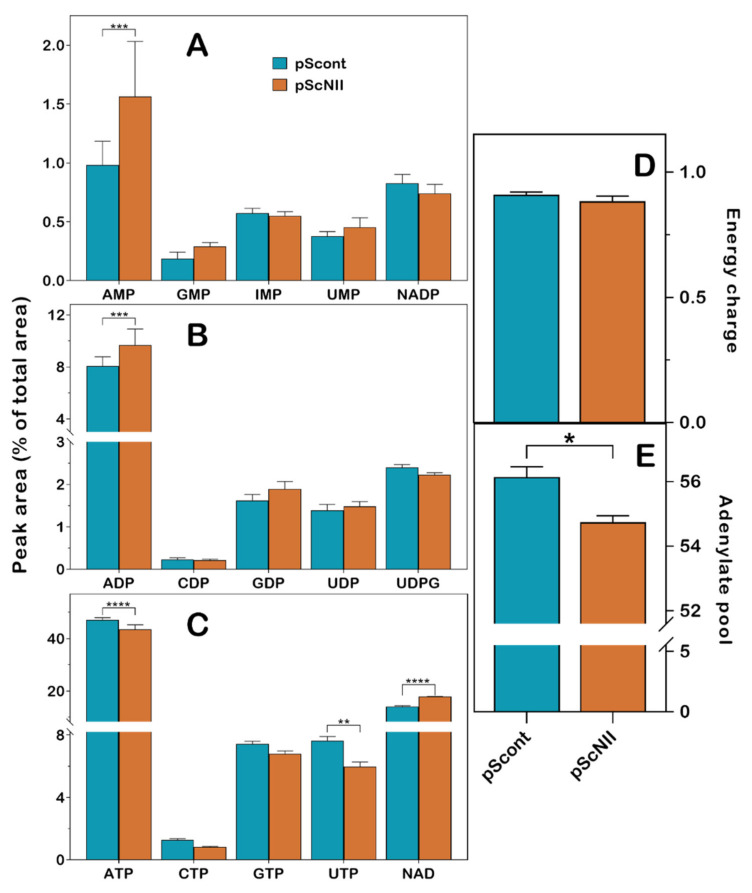
Effect of cN-II silencing on purine and pyrimidine nucleotide content in NCI-H292 cells. (**A**) monophosphate nucleoside and NADP (NADP^+^ plus NADPH) content; (**B**) diphosphate nucleoside content; (**C**) triphosphate nucleoside and total NAD (NAD^+^ plus NADH) content; (**D**) energy charge; (**E**) adenylate pool. Nucleotides were separated by HPCE as described in Materials and Methods. Results are the mean ± SD of three independent experiments and are expressed as a percentage of peak area of each compound normalized to the protein content. Adenylate pool is expressed as the sum of the peak area of each adenylated compound. * *p* < 0.05, ** *p* < 0.01, *** *p* < 0.001, **** *p* < 0.0001.

A comparison of the nucleotide content, determined by high performance capillary electrophoresis (HPCE) analysis, of control and silenced NCI-H292 cells, revealed no significant alteration of the intracellular level of IMP and GMP, the best substrate for cN-II, whereas a significant increase of AMP (1.56 ± 0.47 vs. 0.98 ± 0.20, *p* = 0.0004) concentration was observed (Figure 1A). In addition, ADP (9.68 ± 1.23 vs. 8.06 ± 0.73, *p* < 0.0004) and total NAD (oxidized and reduced) (17.81 ± 0.12 vs. 14.05 ± 0.30, *p* < 0.0001) concentrations increased (Figure 1B,C), whereas ATP (43.50 ± 1.76 vs. 47.11 ± 0.86, *p* < 0.0001) and UTP (5.96 ± 0.31 vs. 7.65 ± 0.26, *p* = 0.0045) were slightly decreased in pScNII cells (Figure 1C). Therefore, cN-II silencing decreased the rate of purine nucleoside monophosphate degradation, leading to the accumulation of AMP and ADP that cannot be readily phosphorylated to ATP. This caused a modest, but significant decrease of the triphosphorylated compounds ATP and UTP. Nevertheless, the energy charge ([ATP] + 0.5[ADP])/([AMP] + [ADP] + [ATP]) was not significantly altered, whereas the total adenylate content (AMP+ADP+ATP) was slightly lowered in silenced cells (Figure 1D,E).

### 2.2. Effect of cN-II Silencing on Proliferation of NCI-H292 Cells

**Figure 2:**
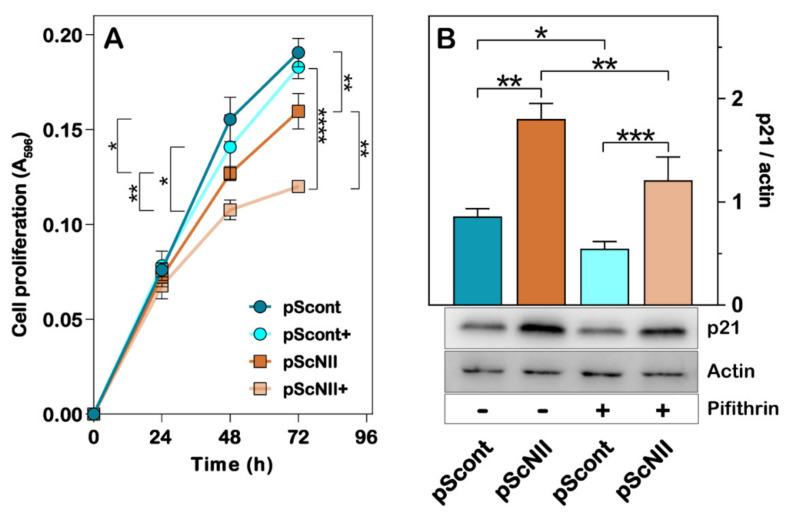
Effect of cN-II silencing on NCI-H292 cell proliferation and p21 expression. (**A**) NCI-H292 pScont and pScNII cell growth with or without 10 µM pifithrin-α was monitored at 0, 24, 48, and 72 h measuring the absorbance at 596 nm of cells stained with crystal violet. Data are the mean ± SD of two experiments performed in sextuplicate. (**B**) The subfigures are representative images of the densitometries shown in the bar graphs for p21. NCI-H292 pScont and pScNII cells were cultured with or without 10 µM pifithrin-α for 48 h and expression of p21 was analyzed as described in Materials and Methods. β-actin was used as loading control. The ratio between the intensity of the p21 and actin bands was calculated and the results are expressed as the mean ± SD of three independent experiments. * *p* < 0.05, ** *p* < 0.01, *** *p* < 0.001, **** *p* < 0.0001.

In a previous paper, we demonstrated that cN-II silencing caused a decrease in the growth rate of a lung carcinoma cell line (A549), and we advanced the hypothesis that activation of p53 was responsible for this occurrence [8]. Similar results were obtained with NCI-H292 cells. Indeed, at 48 and 72 h of growth, pScNII cells had a significantly lower proliferative capacity as compared to control cells (Figure 2A). The presence in the growth medium of pifithrin-α, a well-known p53 inhibitor [23], enhanced the negative effect of cN-II silencing on the proliferative capacity of NCI-H292 cells (Figure 2A). In addition, immunoblot analysis demonstrated that expression of p21, which regulates the cell cycle progression, and is controlled by p53 [24], is higher in silenced cells, whereas the exposure to pifithrin-α decreases the p21 expression (Figure 2B).

**Figure 3:**
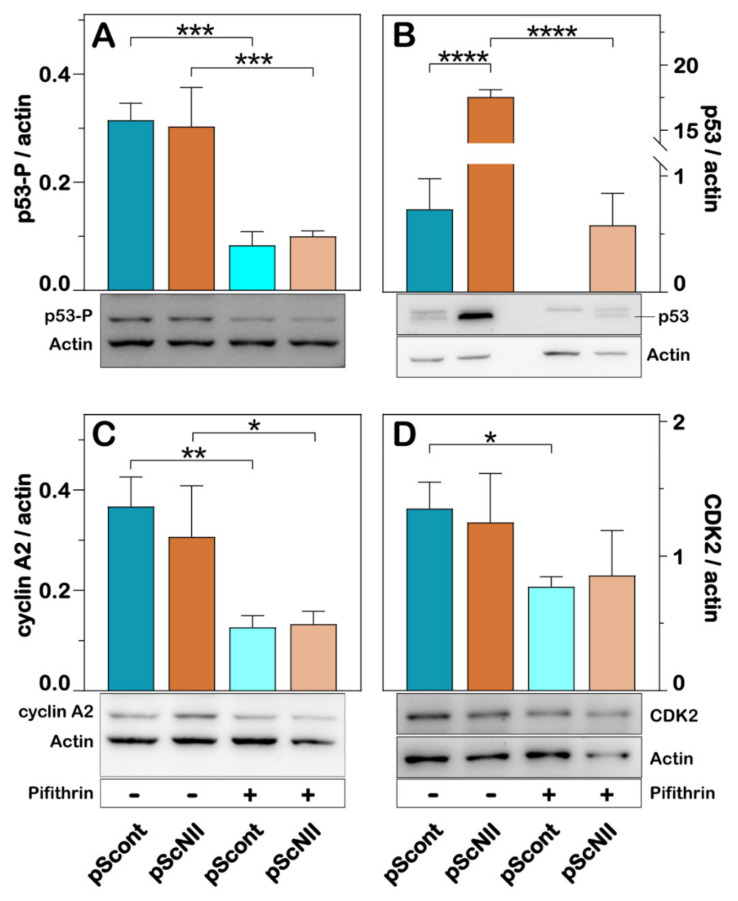
Effect of cN-II silencing on expression of p53, cyclin A2 and CDK2. The subfigures are representative images of the densitometries shown in the bar graphs for phosphorylated p53 (p53-P) (**A**), total p53 (**B**), cyclin A2 (**C**) and CDK2 (**D**). NCI-H292, pScont and pScNII cells were cultured with or without 10 µM pifithrin-α for 48 h and expression of the proteins was analyzed as described in Materials and Methods. β-actin was used as loading control. The ratio between the intensity of the different protein bands and that of actin was calculated and the results are expressed as the mean ± SD of three independent experiments. * *p* < 0.05, ** *p* < 0.01, *** *p* < 0.001, **** *p* < 0.0001.

Expression of p53 was higher in pScNII cells than in pScont cells, whereas both intracellular concentration and phosphorylation were reduced by pifithrin-α (Figure 3A,B). For a better understanding of the molecular mechanisms underlying the observed effect of cN-II silencing on NCI-H292 cell proliferation, we also measured, through immunoblotting, the expression of cyclin A2 and cyclin-dependent kinase 2 (CDK2), which are both known to be involved in the cell cycle progression [25]. No significant difference in the expression of cyclin A2 and CDK2 was found in pScNII cells as compared to pScont. Consistently with the effect exerted by pifithrin-α on NCI-H292 cell growth (Figure 2A), the p53 inhibitor significantly reduced the expression of both proteins (Figure 3C,D).

### 2.3. cN-II Silencing Increases Oxidative Metabolism and Thiol Content in NCI-H292 Cells

**Figure 4:**
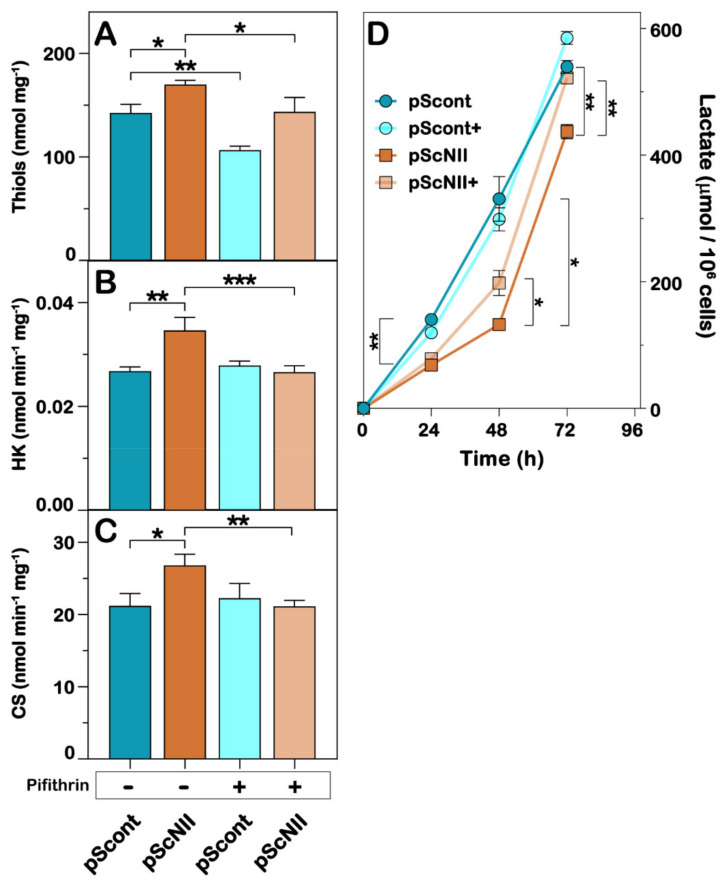
Effect of cN-II silencing on enzyme activities, thiols and lactate levels in NCI-H292 cells. (**A**) Thiols level; (**B**) hexokinase (HK); (**C**) citrate synthase (CS) and (**D**) lactate levels were assayed as described in Materials and Methods. In (A–C), cell extracts were obtained from pScont and pScNII cells incubated for 48 h with or without 10 µM pifithrin-α. In (D) (+) stands for “in the presence of 10 µM pifithrin-α“. Data are the mean ± SD of three independent experiments performed in triplicate for de determination of thiol and enzyme activities and in sextuplicate for lactate. * *p* < 0.05, ** *p* < 0.01, *** *p* < 0.001.

We have previously reported that, in several cell models, cN-II silencing is followed by an increase in oxidative metabolism and of antioxidant defenses [8,9,16]. Therefore, we measured reduced thiols in NCI-H292 cells and found that also in this cell model, they were slightly more abundant in pScNII cells and the incubation with pifithrin-α was able to reduce their level both in pScont and pScNII cells (Figure 4A). To assess a possible effect of cN-II silencing on oxidative metabolism, we also measured the activity of two enzymes involved in glycolysis (hexokinase, Figure 4B) and Krebs cycle (citrate synthase, Figure 4C). cN-II silenced cells displayed significant higher hexokinase and citrate synthase activities with respect to the control cells and the pre-incubation of the cells with pifithrin-α reverted these levels to the ones observed in pScont cells (Figure 4B,C). In order to support a p53-dependent shift towards a more oxidative metabolism in pScNII NCI-H292 cells, we measured the lactate production in the growth medium in the presence and absence of pifithrin-α. The amount of lactate, measured at 24, 48, and 72 h was significantly higher in control cells than in pScNII cells, indicating that, as previously demonstrated in the lung carcinoma cell line A549 [8], cN-II silencing increased oxidative metabolism. Conversely, the amount of lactate produced by pScNII cells in the presence of pifithrin-α was significantly higher than in its absence, and at 72 h was similar to that produced by control cells (Figure 4D).

### 2.4. The AMPK/mTOR Signaling Pathway Is Affected by cN-II Silencing in NCI-H292 Cells

**Figure 5:**
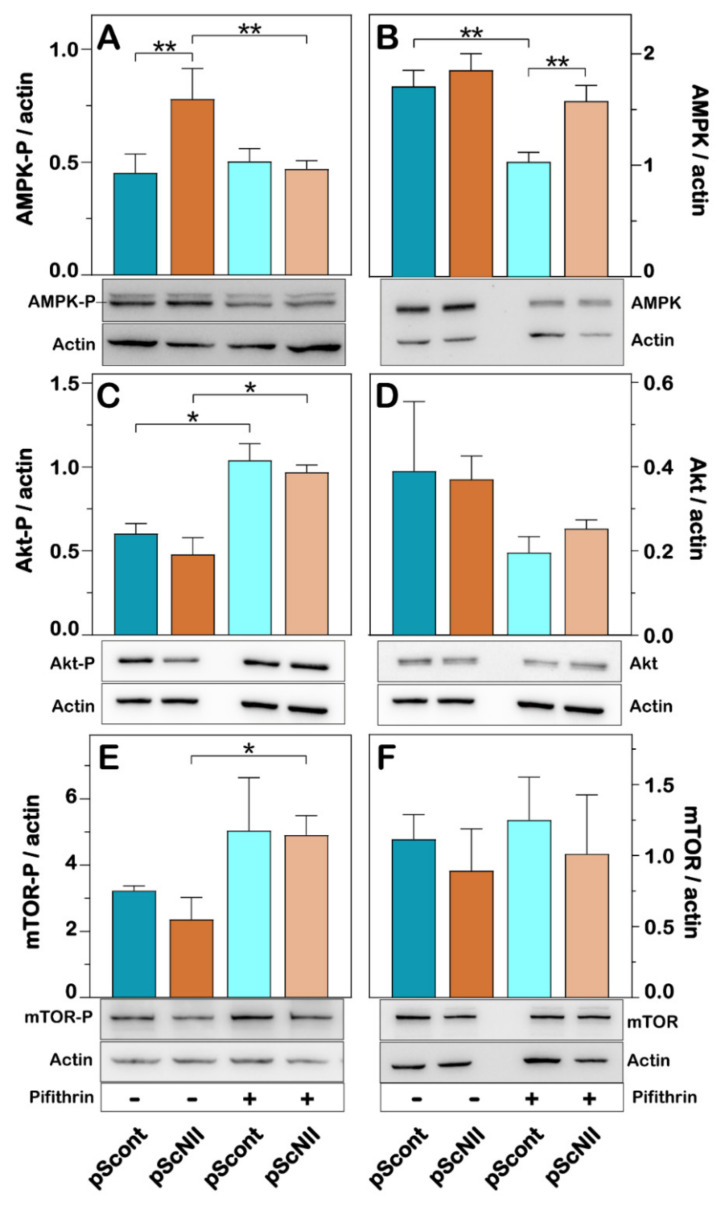
Effect of cN-II silencing on the expression and activation of AMPK, Akt and m-TOR. The subfigures are representative images of the densitometries shown in the bar graphs for AMPK-P (**A**), total AMPK (**B**), Akt-P (**C**), total Akt (**D**), mTOR-P (**E**), and total mTOR (**F**). NCI-H292. pScont and pScNII cells were cultured with or without 10 µM pifithrin-α for 48 h and expression of the proteins was analyzed as described in Materials and Methods. β-actin was used as loading control. The ratio between the intensity of the different protein bands and that of the corresponding actin was calculated and the results are expressed as the mean ± SD of three independent experiments. * *p* < 0.05, ** *p* < 0.01.

Since the AMPK/mTOR signaling pathway plays a central role in the regulation of cellular metabolism [26], we measured the degree of activation of these proteins both in the absence and in the presence of pifithrin-α. Our results indicated that T172-phosphorylated AMPK is more abundant in pScNII cells with respect to the control cells (Figure 5A), although the expression of the protein appears unaffected by cN-II silencing (Figure 5B). The p53 inhibitor caused a decrease of AMPK phosphorylation in pScNII cells to levels comparable of those in pScont cells (Figure 5A).

The expression of total Akt was not affected by cN-II silencing (Figure 5D), whereas the activated Akt (Akt-P), showed a tendency to decrease, in silenced cells compared to the control (Figure 5C). In the presence of pifithrin-α, total Akt expression showed a tendency to decrease (Figure 5D) whereas Akt phosphorylation increased in both cell models. Accordingly, the activated form of mTOR (mTOR-P) showed a tendency to decrease in pScNII cells, whereas in the presence of pifithrin-α, the expression of the phosphorylated mTOR increased significantly in pScNII cells (Figure 5E). No significant difference in the expression of total mTOR was observed in pScont and pScNII cells in the presence and absence of pifithrin-α (Figure 5F).

## 3. Discussion

cN-II is a highly regulated enzyme catalyzing IMP, GMP, and AMP dephosphorylation, thus potentially regulating the intracellular concentrations of purine nucleotides newly synthetized or salvaged [10]. Therefore, it is not surprising that the fluctuation of its expression or activity may influence many cellular mechanisms. In this paper, we demonstrate that the metabolic effects exerted by cN-II partial silencing are not directly correlated with a disturbance of purine nucleotide pool but are mediated by the p53/AMPK signaling pathway. We previously demonstrated that in several tumoral cell models, partial cN-II silencing caused a decrease of the cell growth rate and motility, and an increase in oxidative metabolism and autophagy [8,15]. Theoretically, since cN-II catalyzes the dephosphorylation of IMP and GMP but also of AMP, all the observed effects might be due to an increase in AMP concentration in silenced cells, with the consequent activation of AMPK that exerts a positive effect on mitochondrial proliferation and oxidative metabolism [27]. However, while the described biological effects have been observed in many different cell models, the effect exerted by cN-II silencing on the intracellular nucleotide concentration is more controversial. In fact, in some cases, the intracellular purine and pyrimidine compound levels in cultured cells are not significantly affected by cN-II silencing [15,22,28,29]. In the present paper, we describe that cN-II silencing in a pulmonary carcinoma cell line (NCI-H292) caused a significant increase in AMP and ADP, whereas ATP along with UTP were slightly, but significantly, decreased. We also observed a significant increase in NAD concentration in silenced cells thus confirming that cN-II participates in the metabolism of NAD and in the regulation of its homeostasis [27]. After cN-II silencing in the lung carcinoma cell line A549, we previously demonstrated a significant increase in triphosphorylated and diphosphorylated nucleoside compounds, whereas the nucleoside monophosphates remained unaffected [8]. This difference between the two cell lines might be due to a lower capacity of NCI-H292 cells to phosphorylate ADP, possibly because the cell growth medium contained 10 mM instead of 25 mM glucose present in the A549 growth medium. In fact, when NCI-H292 cells were grown in the presence of 25 mM glucose no significant differences were measured in nucleoside triphosphate pool [22]. Furthermore, NCI-H292 cells, after cN-II silencing, as demonstrated in the present paper, displayed a slower growth rate, but when NCI-H292 were grown at high glucose concentration no differences in proliferation could be measured between pScont and pScNII [22], indicating again that the availability of a consistent amount of energy source plays an important role in determining the effects of cN-II silencing. In pScNII NCI-H292 cells, the decrease of proliferation was accompanied by a higher p21 and p53 protein concentration, whereas p53 phosphorylation was unaffected. The phosphorylation on serine-15 was reported to increase p53 stability through disruption of Mdm2 binding [30,31], therefore the increase of p53 intracellular concentration in pScNII is to be ascribed to a strong increase of protein expression and/or to an alternative stabilization mechanism. The mechanism by which pifithrin-α inhibits p53 activity is still elusive and somehow dependent on cell model. Nevertheless, it was recently demonstrated that this inhibitor is sometimes able to prevent both p53 expression and its translational modifications [32,33]. In our hands, both p53 intracellular concentration and phosphorylation in NCI-H292 cells are inhibited by pifithrin-α as well as the expression of p21 protein, whose gene is a target of p53. However, the p53 inhibitor was unable to revert the decrease of cell proliferation in cN-II silenced cells, suggesting that some toxic effect might be exerted by pifithrin-α on silenced cells. In fact, the proliferation in the presence of the p53 inhibitor was significantly decreased, as well as cyclin A2 and CDK2 expression level, probably in a p53-independent manner. In line with our results, it was recently demonstrated that pifithrin-α could not rescue cell proliferation in tumor cells treated with the disruptor of Mdm2-p53 interaction nutlin-3. Instead, it caused a decrease in cell proliferation, whereas the p53 inhibitor increased proliferation in primary fibroblast cultured in the same conditions [33]. A reduction on cell proliferation exerted by pifithrin-α was also demonstrated in A549 cells [34]. All the metabolic effects such as enzyme expression and lactate production induced by cN-II silencing were reverted by the p53 inhibitor, thus the shift towards a more oxidative phenotype of silenced cells appears to be p53-dependent. In accordance with the described metabolic effect, AMPK was more activated in silenced cells with respect to control cells and pifithrin-α reverted the effect. In conclusion, p53 and a consequent AMPK activation are responsible for the changes in metabolic performances observed after cN-II silencing in NCI-H292 cells. Even though the increase of AMP in cN-II silenced NCI-H292 cells might suggest that AMPK activation is not necessarily dependent on p53, we noticed that the accumulation of AMP in these cells was not very pronounced and, as previously demonstrated, was completely absent in A549 cell line in which the effects of cN-II silencing were similar [8]. Furthermore, incubation with pifithrin-α reverted AMPK activation, which therefore appears to be p53-dependent. Finally, it will be very interesting to find the link between cN-II silencing and p53 activation. Since cN-II can hydrolyze AMP we may expect that cN-II silenced cells not only accumulate AMP, but also have a reduced capacity to release adenosine. In this respect, in a breast cancer cell line (MCF-7), an antagonist of A1 adenosine receptor has been found to cause a significant increase of p53 expression [35]. Besides, cN-II may interact with a plethora of other proteins (for details see the following databases BioGRID; IntAct-EMBL-EBI) including enzymes, intracellular inflammatory receptors [14], transcription factors and even tyrosine kinase receptors, presumably with relevant regulatory functions [9]. Therefore, we suggest that the level of cN-II expression might be relevant in determining protein-protein interactions with consequences on both cN-II and interacting protein activities possibly interfering with mechanisms involved in p53 expression and function. On the contrary, we failed to demonstrate that the change in proliferation in cN-II silenced cells were p53-dependent, therefore more investigations are required to understand the molecular mechanisms underlying this observation.

Our results are casting a new light on the function of cN-II in tumor cells and its role in chemoresistance. cN-II overexpression was indeed associated with chemoresistance to several drugs even those that were not substrate of enzyme hydrolyzing activity [36]. The inverse relationship between cN-II and p53 expression indicates that cells expressing high cN-II activity are more aggressive and therefore have a lower sensitivity to the chemotherapeutic drugs independently of the cN-II ability in decreasing the intracellular concentration of the active drug. Moreover, mutations in cN-II codifying gene or in the flanking sequences have been associated with a number of psychiatric and psychomotor disturbances, including schizophrenia Parkinson’s disease and spastic paraplegia [20]. While this association is demonstrated by several reports, the causal relationship between the lack of the protein and or of its enzyme activity and the diseases, is still waiting to be clearly described. In this paper, we investigated the consequence of cN-II silencing as a model of a lack of both protein and activity. Our results indicate an interesting causal relationship between enzyme expression and p53 activation that may have dangerous consequences in a developing organism. New experiments, both on additional cell lines and with animal models, could be performed in order to confirm our findings.

## 4. Materials and Methods

### 4.1. Materials

Protease inhibitor cocktail, ATP, 2,3-bisphosphoglyceric acid, acetyl-CoA, ethylenediaminetetraacetic acid (EDTA), D-glucose, phenylmethyl sulphonyl fluoride (PMSF), 5,5′-dithiobis(2-nitrobenzoic acid) (DTNB), sodium fluoride, sodium orthovanadate, β-glycerophosphate, sodium pyrophosphate, dimethylsulfoxide (DMSO), perchloric acid, glucose-6-phosphate dehydrogenase (G6PDH), pyruvate, oxaloacetate, NADH, NADP^+^, crystal violet, 2,2′-azino-di-[3-ethylbenzothiazoline-sulfonate] (ABTS), l-lactate oxidase, horseradish peroxidase, and pifithrin-α were from Sigma (Milano, Italy); [8-^14^C]-inosine was from Moravek Biochemicals and Radiochemicals (Brea, CA, USA); RPMI medium, penicillin, streptomycin, fetal bovine serum (FBS), glutamine and trypsin were from Euroclone (Pero, Milan, Italy). DE-81 chromatographic paper was from Whatman (Madstone, UK); scintillation liquid Optiphase Hisafe 2 was from PerkinElmer (Waltham, MA, USA). Chemiluminescence Detection System and polyvinylidene fluoride (PVDF) membranes were from Millipore (Burlington, Massachusetts, USA); primary antibodies specific for AMPK-P (T172) (#2535) or Akt(pan) (#2920), Akt-P (Ser473) (#4060), AMPK (#2795), p53- p(Ser15) (#9286), p53 (#2524), β-Actin (#8457), mTOR (#4517), mTOR- P(Ser2448),Cyclin A2/#4656), CDK2 (#2546), p21 (#2947), horse radish peroxidase (HRP)-linked anti-mouse (#7076) and anti-rabbit IgG (#7074) were from Cell Signaling (Danvers, MA, USA). All other chemicals were of reagent grade.

### 4.2. Cell Culture and Cell Proliferation Assay

Mucoepidermoid pulmonary carcinoma cell lines (NCI-H292) partially silenced (pScNII) with the respective control (pScont) were developed by stable transfection of shRNA as indicated in [22]. The cell lines were routinely tested for *Mycoplasma* contamination by PCR. Human NCI-H292 cells were cultured in RPMI containing 10 mM glucose, 10% FBS, 1% glutamine, 100 U/mL penicillin and 100 U/mL streptomycin (standard medium). The cells were cultured in an incubator with 5% CO_2_ at 37 °C. Cell proliferation assay was performed by the crystal violet staining method [37]. Briefly, 12 h before treatment, four 24 multi-well culture plates were seeded with 0.8 mL of standard medium containing 20,000 cells/well. When indicated, cells were exposed to 10 µM pifithrin-α and after 0, 24, 48 and 72 h of incubation, the medium was removed and the cells were stained with 0.1% crystal violet solution in methanol for 30 min at 37 °C under gentle shaking. Then, the plates were carefully washed three times in distilled water and dried. Acetic acid (0.6 mL, 10%) was added to the wells and kept 15 min at room temperature under gentle shaking. One hundred microliters from each well were transferred into a 96 multi-well for the quantitative analysis by absorbance measurements at 596 nm in an automatic ultra-microplate reader EL 808 Bio-Tek Instruments. Inc. (Winooski, VT, USA).

### 4.3. High Performance Capillary Electrophoresis (HPCE) Analysis

NCI-H292, pScont and pScNII cells were plated at a density of 3.3 × 10^4^/cm^2^ in 10 mL medium in 100 mm diameter plates. After 12 h, medium was withdrawn and replaced with 7 mL of the same medium (3 plates for each cell type). After 48 h, plates were washed with phosphate buffer saline (PBS), cells were trypsinized, rapidly separated from medium by centrifugation (400× *g* for 5 min) and resuspended in 170 μL 1 M trichloroacetic acid (TCA). To avoid degradation of nucleotides, samples were treated as previously described [38,39]. After three rounds of rapid freezing, pellets were separated from supernatants by centrifugation (14,000× *g* for 1 min). One hundred and twenty μL of each supernatant was collected and immediately back-extracted three times with 1.2 mL ether. Samples were kept at room temperature under a chemical hood for 5 min and later stored at −20 °C until needed. The protein content of the pellets previously precipitated with 1 M TCA was determined as described by Peterson [40]. The HPCE analysis was performed on the supernatants. All the experiments were performed using a Beckman P/ACE MDQ Capillary Electrophoresis System (Pasadena, CA, USA) equipped with a diode array detector as previously described [38]. The elution profile was followed at 254 nm.

### 4.4. Quantification of Reduced Thiols

Reduced thiol level was determined following Ellman method with some modifications [41]. Cells were plated at a density of 3.3 × 10^4^/cm^2^ in 7 mL of standard medium in 100 mm diameter plates. After 12 h, medium was withdrawn and replaced either with 7 mL of the same medium (control samples, 3 plates for each cell type) or with 7 mL of medium containing 10 µM pifithrin-α (3 plates for each cell type). After 48h, cells were washed with PBS, trypsinized and resuspended in 100 mM Tris-HCl pH 7.4 (400 μL) in the presence of protease inhibitor cocktail. Cell lysates were obtained by three freeze/thaw cycles at −80 °C, followed by centrifugation at 10,000× *g* for 40 min at 4 °C. Sixty three μL of the supernatant, referred to as crude extract, was added to 7 μL of 6 N perchloric acid, strongly shacked and centrifuged 5 min at 10,000× *g*. For the determination of reduced thiol content, 5 μL of deproteinized extract was added to a solution of 0.5 mM DTNB dissolved in 1% sodium citrate, 250 µM EDTA, 250 mM sodium phosphate buffer pH 7.4 in a total volume of 700 μL. The absorbance at 412 nm was measured using a Beckman (Pasadena, CA, USA) spectrophotometer. The reduced thiol levels were calculated using an extinction coefficient of 13,600 M^−1^ cm^−1^.

The protein content of the crude extract was determined following the method described by Bradford [42].

### 4.5. Enzymatic Assays

Cells plated and treated in the presence and in absence of 10 µM pifithrin-α as described for quantification of reduced thiols were washed with PBS, trypsinized and resuspended in 400 μL of 100 mM Tris-HCl pH 7.4 in the presence of protease inhibitor cocktail, and 10 mM NaF, 30 mM β-glycerophosphate, 10 mM sodium-pyrophosphate and 2 mM sodium-orthovanadate as phosphatase inhibitors. Crude extract, obtained as described in the previous paragraph was collected and protein content determined.

Hexokinase activity was measured following the method described by Gerber [43], with some modifications. The assay was carried out by continuous spectrophotometric monitoring the absorbance at 340 nm at 30 °C (extinction coefficient of 6220 M^−1^ cm^−1^). The enzyme assay mixture contained: 50 mM Tris-Cl pH 7.4, 120 mM D-glucose, 10 mM MgCl_2_, 0.6 mM ATP, 0.25 mM NADP^+^, 0.04 Units G6PDH and 100 µg of crude extract in a total volume of 800 µL.

Nucleoside phosphotransferase activity of cN-II was measured by determining the nucleoside monophosphate synthesized in the presence of the labeled nucleoside [44]. Incubations were performed at 37 °C in a medium containing 4.5 mM ATP, 20 mM MgCl_2_, 2 mM IMP, 100 mM Tris-HCl, pH 7.4, 1 mM [8-^14^C]-inosine (3500 dpm/nmol) and 10 μg of crude extract in a total volume of 50 μL. The reactions were stopped by spotting 10 μL of the incubation medium on DE-81 paper disks followed by washing 15 min in 1 mM ammonium formate and 10 min in two changes of water. The disks were dried and placed in counting vials filled with 8 mL of Optiphase Hisafe scintillation liquid. The enzymatic activities were calculated from the amount of radioactive nucleoside monophosphate formed.

Citrate synthase assay was performed according to [8]. The assay was carried out by continuous spectrophotometric monitoring of the change in absorbance of DTNB at 412 nm at 30 °C (extinction coefficient of 13,600 M^−1^cm^−1^). The enzyme assay mixture contained 80 mM Tris-Cl pH 8.3, 0.05 mM acetyl-CoA, 0.5 mM oxaloacetate, 80 µM DTNB and 30 μg of crude extract in a total volume of 800 µL.

One enzyme unit is the amount of enzyme that catalyzes the conversion of 1 µmol of substrate/min in the described conditions.

GraphPad Prism Ver. 8 (GraphPad Software, San Diego, CA, USA) was used to estimate the kinetic parameters, using a hyperbolic nonlinear regression analysis.

### 4.6. Western Blotting

Cells were plated as described in 4.2, and incubated both in the presence and in absence of 10 µM pifithrin-α. After 48 h incubation, cells were washed with 1 mL cold PBS in the presence of protease inhibitor cocktail, supplemented with 1 mM PMSF, 1 mM sodium orthovanadate, 5 mM sodium pyrophosphate, 20 mM β-glycerophosphate. The medium was then removed, cells were scraped off and collected. Plates were then washed with 1 mL cold PBS containing protease inhibitors and remaining cells collected and added to the previous ones, then centrifuged at 700× *g* for 5 min at 4 °C. Supernatants were discarded and 250 μL of lysis buffer containing 150 mM NaCl, 50 mM NaF, 0.5 mM EDTA pH 8.0, 0.5% Triton X-100, 1 mM PMSF, 1 mM sodium orthovanadate, 5 mM sodium pyrophosphate, 20 mM β-glycerophosphate in 25 mM Tris-Cl pH 7.4, was added. Vials with cells and lysis buffer were then strongly shacked for 1 min and kept on ice for 10 min prior to centrifugation at 10,000× *g* for 40 min at 4 °C. The supernatants were collected and concentration of protein extracts was determined. Protein samples (30 μg each lane, 40 μg for p53 protein), were resolved by 12% SDS-PAGE at 200 V for 45 min and transferred onto PVDF membranes using Trans Blot Turbo Transfer system (Bio-Rad). Membranes were blocked with TBSTa (50 mM Tris-HCl pH 7.5 supplemented with 150 mM NaCl, 0.1% (*v/v*) Tween-20 and 5% (*w/v*) skim milk powder) or TBSTb (50 mM Tris-HCl pH 7.5 supplemented with 150 mM NaCl, 0.1% (*v/v*) Tween-20 and 5% (*w/v*) bovine serum albumin) for 1 h. Membranes were incubated with primary antibody overnight at 4 °C, then secondary antibody was added and kept for 1 h at room temperature before visualizing chemiluminescence of protein bands using ChemiDoc imaging system (Bio-Rad). Primary antibodies specific for Akt (pS473) 1:1000 in TBSTb, AMPK-P (T172) 1:500 in TBSTb, p53-P (S15) 1:500 in TBSTa, p53 1:500 in TBSTa, Akt (pan) 1:2000 in TBSTb, AMPK 1:500 in TBSTa, Cyclin A2 1:2000 in TBSTa, CDK2 1:1000 in TBSTb, mTOR 1:1000 in TBSTa, mTOR-P (pS2448) 1:1000 in TBSTb, p21 1:1000 in TBSTa, β-actin 1:2000 in TBSTa and HRP-linked secondary antibodies anti-mouse and anti-rabbit IgG 1:1000 in TBSTa were used. Relative abundance of proteins was determined using Bio Rad Image Lab version 6 software.

### 4.7. Lactate Concentration Measurement

The lactate concentration was determined in the medium of pScont and pScNII cells grown as described in 4.2. In the presence and absence of 10 µM pifithrin-α. At 0, 24, 48, and 72 h incubation, 500 µL of the medium were withdrawn and centrifuged 5 min at 10,000× *g*. Two µL of the supernatant was used for the assay as described by Liaud et al. [45]. The colorimetric detection of lactate exploits two sequential oxido-reduction reactions. The first one is the conversion of L-lactate and O_2_ into pyruvate and H_2_O_2_, catalyzed by the stereospecific l-lactate oxidase. In the second reaction, catalyzed by HRP, the chromogenic substrate ABTS is oxidized by H_2_O_2_ produced in first reaction. The oxidized ABTS absorbs at 420 nm and dyes the reaction mixture in green. The ABTS oxidation is proportional to L-lactate concentration which can be calculated from a standard curve of known L-lactate concentration.

### 4.8. Statistical Analyses

All data are reported as the mean ± SD. Significant differences among groups were determined using Two-way analysis of variance (ANOVA) followed by Tukey multiple comparison test with GraphPad GraphPad Prism Ver. 8 (GraphPad Software, San Diego, CA, USA). A *p*-value < 0.05 was considered to indicate statistical significance.

## Figures and Tables

**Table 1:** cN-II activity (mU/mg) in NCI-H292 cells.

NCI-H292 Cell Line	with Pifithrin-α	without Pifithrin-α
pScont	2.3 ± 0.1	2.0 ± 0.2
pScNII	0.7 ± 0.03 ****	0.8 ± 0.01 ****

cN-II activity was measured as described in Methods, in cells grown both in the absence or in the presence of 10 µM pifithrin-α. Results are the mean ± SD of three independent experiments. **** *p* ˂ 0.0001 (pScNII vs. pScont).

## Data Availability

The data that support the findings of this study are available from the corresponding author upon reasonable request.
